# Simultaneous frontal and orbital abscess rare complications of otogenic origin: Case report and literature review

**DOI:** 10.1016/j.amsu.2022.103458

**Published:** 2022-03-07

**Authors:** Abdelkouddous Laaidi, Marouane Makhchoune, Yassin Tahrir, Mohamed Yassine Haouas, Abdessamad Naja, Abdelhakim Lakhdar

**Affiliations:** Neurosurgery Department, University Hospital Center IBN ROCHD, Casablanca, Morocco

**Keywords:** Abscess, Surgery, CT, SCAN, Antibiotic, Case report

## Abstract

Otogenic frontal abscess is an uncommon location of otogenic abscess of the brain and constitutes less than 5%. And the orbital extension is even more exceptional. An 11-year-old child, presenting with a two-week-long history of an acute otitis badly treated. Admitted for headaches, fever, vomiting and left eyelid swelling. The preoperative CT scan revealed a left frontal epidural abscess associated to a sub-periosteal Abscess. The patient was operated on. A supraorbital incision through the eyebrows allowed the evacuation of the periorbital abscess and the cerebral empyema through a trephine hole. The patient received probabilistic intravenous antibiotic therapy with ceftriaxon, aminoglycoside and metronidazole. Then relay per os. Postoperative recovery was marked by disappearance of headaches at postoperative Day two and the periorbital edema at day six. The patient was discharged home at postoperative week four with oral antibiotic therapy. Three months postoperative months follow-up CT scan revealed a total radiological cleaning. Otogenic frontal abscess associated to orbital Abscess is extremely rare and should be considered in front of ophthalmological signs. The management is multidisciplinary, and the entry point treatment mustn't be forgotten.

## Highlights

• This paper presents an 11-year-old boy complaining presenting with a two-week-long history of an acute otitis badly treated. Admitted for headaches, fever, vomiting and left eyelid swelling. The neurological examination found a conscious child GCS scores were E4V5M6 with no deficit, and palpebral edema.
• The CT scan revealed a left frontal epidural abscess associated to a sub-periosteal Abscess. A supraorbital incision through the eyebrows allowed the evacuation of the periorbital abscess.
• Otogenic frontal abscess associated to orbital Abscess should be considered in front of ophthalmological signs. The management is multidisciplinary, and the entry point treatment mustn't be forgotten.

## 1 Introduction

Otogenic brain abscesses are one of the most serious complications of otological infections. They are commonly located in the temporal lobe or cerebellum. Frontal lobe seems to be an uncommon site of otogenic abscess of the brain and constitutes only 5% [4]. And the orbital extension is even more exceptional. We report in our paper an unusual case of otogenic frontal abscess associated to orbital abscess treated by surgical evacuation with a supraorbital incision through the eyebrows. Also, we conducted a literature review to focus on the pathological mechanisms and principles of management of this rare location at the light of the previously reported cases.

Most papers specifically addressed the intracranial location of abscesses of otogenic infection. Out of 1302 total otogenic abscesses, 55% (n 5722) were found in the temporal lobe and 28% (n 5369) were in the cerebellum. Other locations mentioned included the frontal and pari-et al. lobes, which were implicated in 5% (n 5 66 Most papers specifically addressed the intracranial location of abscesses of otogenic infection. Out of 1302 total otogenic abscesses, 55% (n 5722) were found in the temporal lobe and 28% (n 5369) were in the cerebellum. Other locations mentioned included the frontal and pari-et al. lobes, which were implicated in 5% (n 5 66 Most papers specifically addressed the intracranial location of abscesses of otogenic infection. Out of 1302 total otogenic abscesses, 55% (n 5722) were found in the temporal lobe and 28% (n 5369) were in the cerebellum.

## 2 Case report

We report the case of an 11-year-old child, presenting with a two-week-long history of an acute otitis badly treated. Admitted for sudden onset headaches, with fever, vomiting and left eyelid swelling. The neurological examination demonstrated a conscious patient with no deficit, and palpebral edema. There is no history suggestive of any mental or physical illness.

**Fig. 1:**
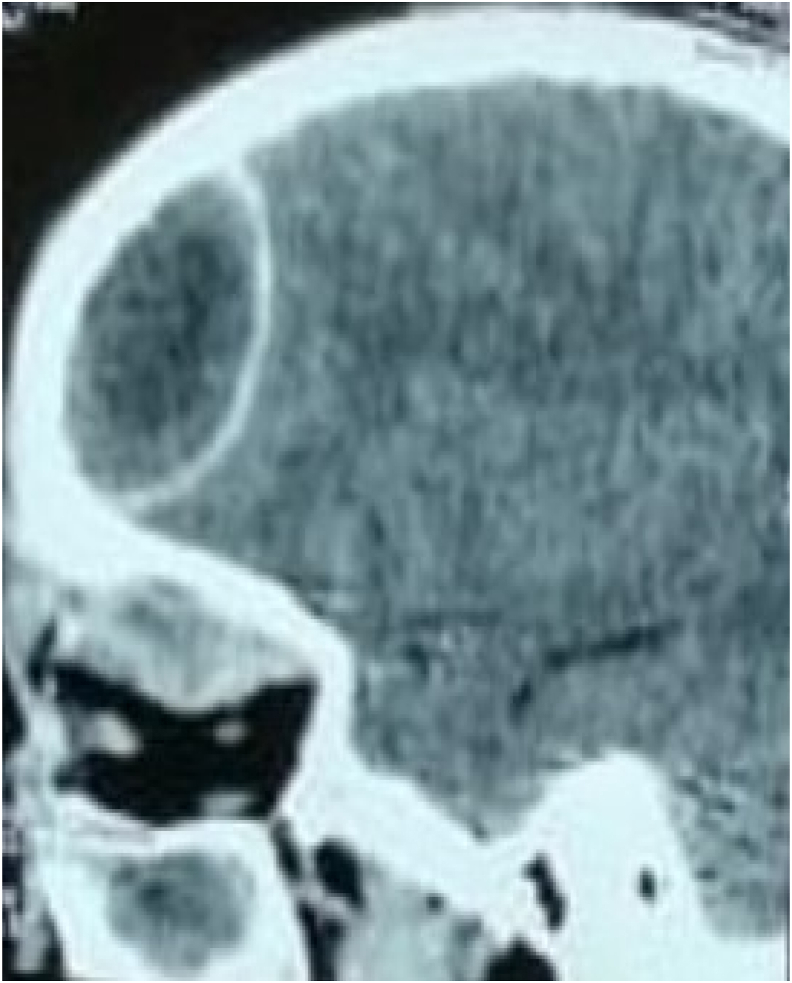
CT scan imaging showing left frontal epidural abscess with enhancement of the lesion's rim after addition of contrast material with an orbital abscess.

The preoperative CT scan revealed a left frontal hypodense collection, with enhancement of the lesion's rim after addition of contrast material with an orbital abscess. The left frontal sinus was not pneumatized. A diagnosis of frontal epidural abscess was made with a sub-periosteal Abscess (Fig. 1).

The intervention was performed by our chief resident under general anesthesia. A supraorbital incision through the eyebrows allowed the evacuation of the periorbital abscess and the cerebral empyema through a trephine hole. 55 cc of pus was evacuated, it was greenish, purulent and foul-smelling.

The patient received probabilistic antibiotic therapy with ceftriaxon, aminoglycoside and metronidazole while waiting for the results of the antibiogram. The direct examination and culture were negative because of the antibiotic treatment for otitis. Intravenous treatment is maintained at 5 days for aminoside, 10 days for metronidazole and 21 days for Ceftriaxon, then relay per os for 6 weeks, also corticosteroids were administrated for cellulitis end cerebral edema.

**Fig. 2:**
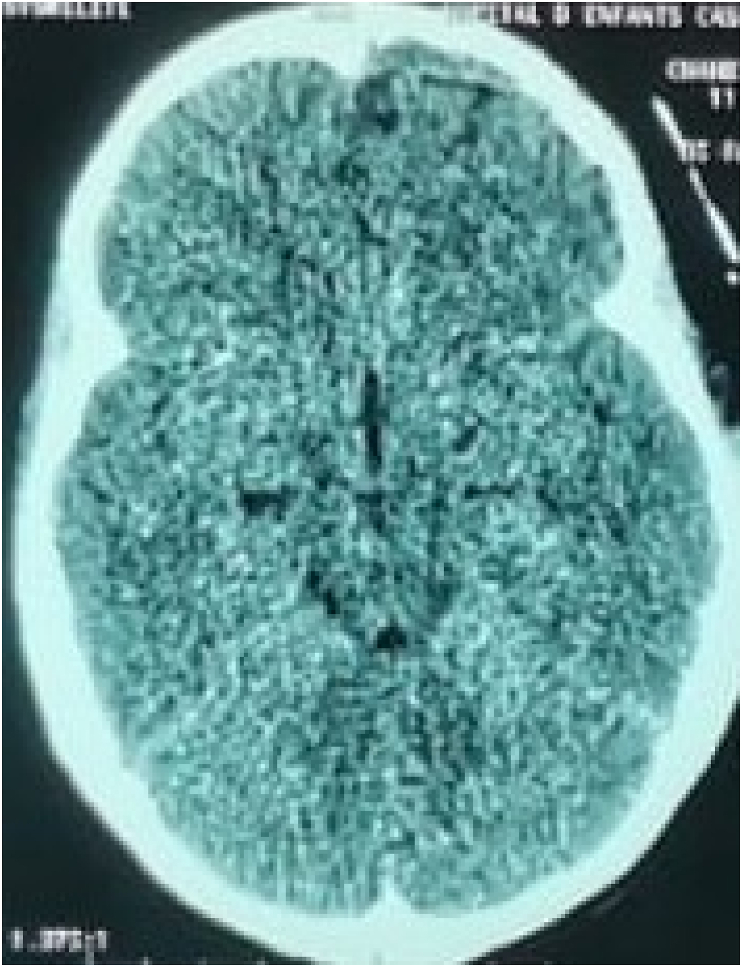
Control CT scan showing regression of frontal and intraorbital abscesses.

The follow-up was marked by disappearance of headaches at postoperative day two and the periorbital edema at day six. Postoperative CT scan is performed on day 21, revealed a clear improvement (Fig. 2).

He was discharged to home at postoperative week four with oral antibiotic therapy.

This case has been reported in line with the 2020 SCARE guidelines [1].

## 3 Discussion

Epidural abscesses constitute 5%–25% of all localized intracranial infections. Various causes of epidural abscesses have been reported and described in the literature, and include paranasal sinusitis, and infection of a prior craniotomy site [2,6]. Approximately 60%–90% of epidural abscesses are related to otitis, mastoiditis, and sinusitis. Other causes include osteomyelitis, rarely associated with Pott puffy tumor [3,7]. Less common causes include orbital suppurations, congenital dermal sinus, and dental infection. Otogenous abscess of the brain is commonly located in the temporal lobe (55%) or cerebellum (28%), frontal location is rare and constitute less than 5% [3,4]. Intra-orbital extension is very rare in this case, and weakly reported in the literature.

Drug and alcohol abuse as well as diseases and treatments leading to immunodepression are important risk factors. It can also be seen after head trauma associated with skull fractures or as a complication of neurosurgical procedures such as craniotomy, skull traction for cervical fractures or scalp venous catheters in children [8].

The most common presenting symptoms of otogenic brain abscess are fever, headache, nausea and vomiting. Also altered mental state, seizures, hemiparesis, and cranial nerve deficits. Papilledema, and meningeal irritation [9].

Periorbital cellulitis and frontal scalp or facial swelling may be present, especially if there is an underlying osteomyelitis. When the abscess arises following an episode of sinusitis or mastoiditis, the early symptomatology is usually related to the primary disease. Some patients may have a history of recently treated upper respiratory tract infection [5,6].

The diagnosis is based on clinical and radiological examinations. cerebral mri remains more precise than CT scan. Some studies have demonstrated that the sensitivity of mri to detect epidural abscesses is superior to that of CT scan [10]. MRI imaging will help to distinguish epidural from subdural collection and is also valuable in establishing communication with contiguous extracranial sites [9,10]. The scanner is more efficient than the MRI when there is a bone involvement and more available than MRI so the most used for surgical planning. In our case the CT scan was sufficient to make the diagnosis and for the postoperative follow-up [11]. A blood culture is found to be positive in approximately 10% of cases [12]. In our case, the culture was negative. In epidural abscess the infection is mainly of polymicrobial origin and anaerobic and aerobic species of streptococci are the most incriminated [12].

Global management involves surgical drainage of the abcess with eradication of the primary site of infection followed by a course of appropriate intravenous antibiotics. We usually start with a broad-spectrum antibiotic therapy against cocci and aerobic and anaerobic bacilli that cross the hematoencephalic barrier [13]. When the antibiogram is determined the antibiotic is adapted according to the result, if the culture is negative the broad-spectrum antibiotic therapy is kept for 4 weeks followed by enteral or parenteral supplementation for up to 8 weeks [13,14]. In our case, we evacuated the epidural and orbital abscesses and the patient has received broad-spectrum antibiotic therapy. In the case of small abscesses with a neurologically intact patient, we opt for medical treatment with clinical and radiological surveillance [14]. The particularity of our case is that the frontral abcess is of otogenic origin with intraorbital extension.

## 4 Conclusion

Otogenic frontal abscess associated to orbital Abscess is extremely rare and should be considered in front of ophthalmological signs. The management is multidisciplinary, and the entry point treatment mustn't be forgotten.

## Financial disclosure

The authors declared that this study has received no financial support.

## Ethical approval

Written informed consent was obtained from the parents for publication of this case report and accompanying images. A copy of the written consent is available for review by the Editor-in-Chief of this journal on request.

## Provenance and peer review

Provenance and peer review.

Not commissioned, externally peer-reviewed.

## Declaration of competing interest

The authors of this article have no conflict or competing interests. All of the authors approved the final version of the manuscript.
